# Age-specific effectiveness of a tuberculosis screening intervention in children

**DOI:** 10.1371/journal.pone.0264216

**Published:** 2022-02-18

**Authors:** Meredith B. Brooks, Melanie M. Dubois, Amyn A. Malik, Junaid F. Ahmed, Sara Siddiqui, Salman Khan, Manzoor Brohi, Teerath Das Valecha, Farhana Amanullah, Mercedes C. Becerra, Hamidah Hussain

**Affiliations:** 1 Department of Global Health and Social Medicine, Harvard Medical School, Boston, Massachusetts, United States of America; 2 Division of Infectious Diseases, Boston Children’s Hospital, Boston, Massachusetts, United States of America; 3 Harvard Medical School, Boston, Massachusetts, United States of America; 4 Yale Institute for Global Health, New Haven, Connecticut, United States of America; 5 Interactive Research and Development Global, Singapore, Singapore; 6 The Indus Hospital and Health Network, Korangi Crossing, Karachi, Pakistan; 7 Sayed Abdullah Shah Institute of Medical Sciences Sehwan, Sehwan, Pakistan; 8 Communicable Diseases Control, Department of Health, Sindh, Hyderabad, Pakistan; Fundació Institut d’Investigació en Ciències de la Salut Germans Trias i Pujol, Universitat Autònoma de Barcelona, SPAIN

## Abstract

**Objective:**

To apply a cascade-of-care framework to evaluate the effectiveness—by age of the child—of an intensified tuberculosis patient-finding intervention.

**Design:**

From a prospective screening program at four hospitals in Pakistan (2014–2016) we constructed a care cascade comprising six steps: screened, positive screen, evaluated, diagnosed, started treatment, and successful outcome. We evaluated the cascade by each year of age from 0 to 14 and report the age-specific mean proportion and standard deviation.

**Results:**

On average across all ages, only 12.5% (standard deviation: 2.0%) of children with a positive screen were not evaluated. Among children who had a complete evaluation, the highest percentages of children diagnosed with tuberculosis were observed in children 0–4 (mean: 31.9%; standard deviation: 4.8%), followed by lower percentages in children 5–9 (mean: 22.4%; standard deviation: 2.2%), and 10–14 (mean: 26.0%; standard deviation:5.4%). Nearly all children diagnosed with tuberculosis initiated treatment, and an average of 93.3% (standard deviation: 3.3%) across all ages had successful treatment outcomes.

**Conclusions:**

This intervention was highly effective across ages 0–14 years. Our study illustrates the utility of applying operational analyses of age-stratified cascades to identify age-specific gaps in pediatric tuberculosis care that can guide future, novel interventions to close these gaps.

## Introduction

Tuberculosis (TB) remains a top-ten killer of children younger than five years [1], reflecting stagnant high rates of TB around the world [2]. Children face a disproportionate gap in TB case detection; while one third of overall TB cases globally are missed every year [2], among children under the age of 15, a staggering two-thirds of cases go unreported or undiagnosed [3–5]. Most of the children who die from TB every year are never even diagnosed [1]. TB diagnosis in children is complicated by two major factors. First, children often present with non-specific symptoms, such as cough, fever, fatigue, poor appetite, and weight loss [6]. Second, children often have paucibacillary TB, which limits the sensitivity of conventional bacteriologic tests for disease. Also, children are often unable to even produce a sputum sample, precluding any potential bacteriologic confirmation of disease. Together, these complications may lead to delays in diagnosis or a misdiagnosis.

To close this deadly case-detection gap, strategies and algorithms must be designed and optimized for specific sub-populations. Where community TB rates are high, children who reach any hospital for any reason, for example, are a key group to screen for TB. Indeed, a child-specific algorithm of intensified patient finding (intensified case finding) can be designed; this is a form of active case finding [7]. Typically an active patient-finding intervention for TB will include a screening step and a diagnostic step; it should be designed according to the characteristics of the target group.

One key quality dimension of a chosen patient-finding intervention is its effectiveness, which will depend on the design of the algorithm and the tests used. Effectiveness should encompass patient retention across all sequential stages of the care cascade [8, 9], and there will be challenges specific to the target group. Among children, the difficulty in obtaining microbiological confirmation of TB disease is paramount and varies by the age of the child [10]. Thus, it is critical to assess effectiveness of patient-finding interventions with a care-focused lens that integrates information about the intervention and about the target group. Notably, there are no published reports assessing pediatric TB patient-finding interventions by age. We sought to evaluate whether a TB patient-finding intervention varied in effectiveness according to the age of the child screened in an intensified, multicenter patient-finding intervention.

## Materials and methods

### Study setting

**Fig 1:**
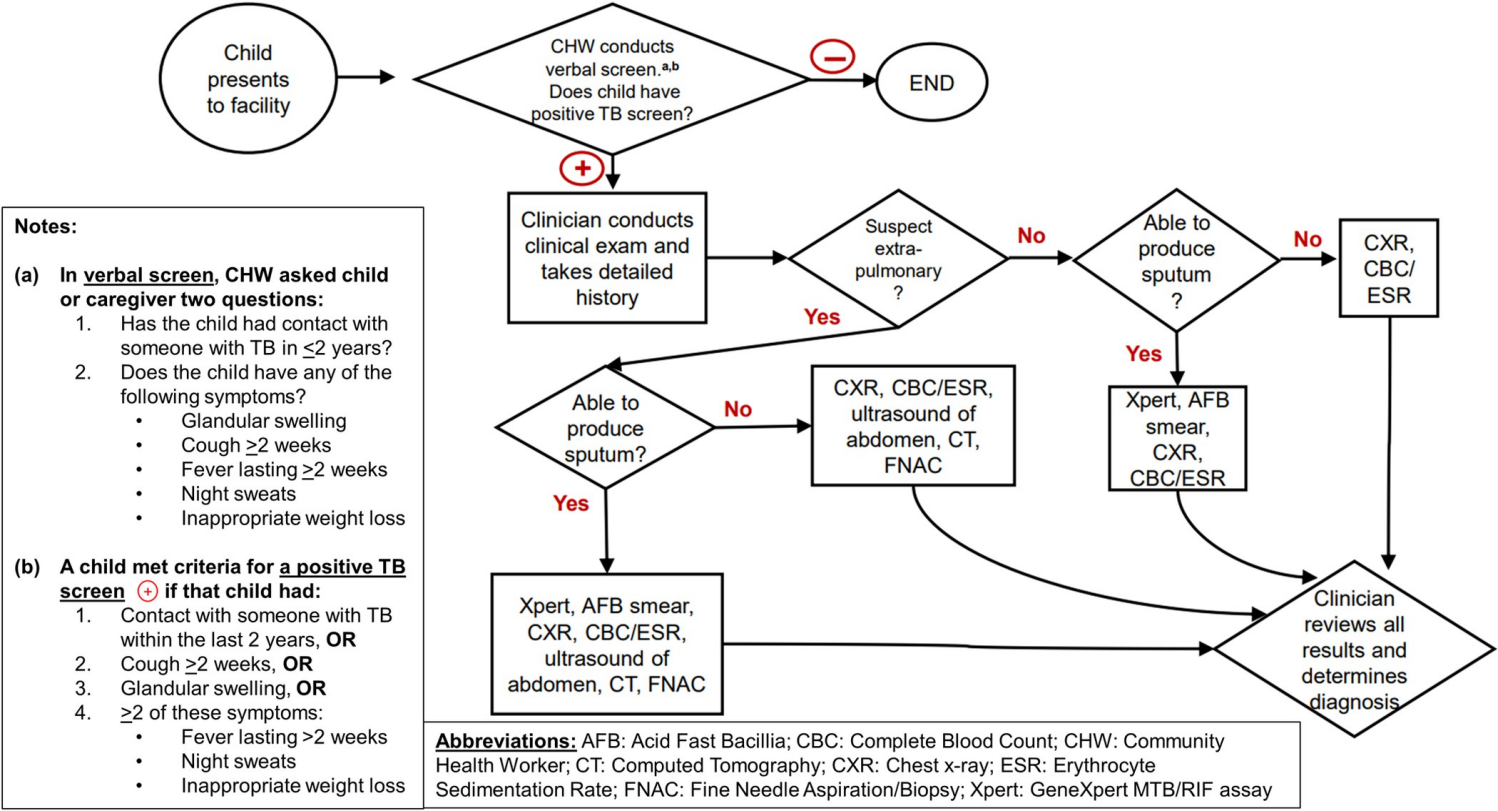
Process map for facility-based intervention to detect TB in children.

Between October 2014 and March 2016, the Indus Hospital and Health Network implemented an intensified TB patient-finding intervention in a rural setting in Pakistan (Fig 1) [11, 12]. Health workers screened each child and caregiver visiting the general, pediatric, and chest outpatient departments at four participating public sector health facilities in Jamshoro District. This screening step consisted of health workers asking whether the child had: (1) cough for two weeks or longer, (2) contact with someone with TB in the last two years, (3) glandular swelling, and/or (4) whether any of the following symptoms were present: fever lasting two or more weeks, night sweats, or inappropriate weight loss. If a child screened positive (defined below in **Step 2. Positive Screen** in the Data collection and measures section) they were referred to a medical officer for further evaluation. Children received a clinical evaluation, including chest X-ray, complete blood count, and erythrocyte sedimentation rate, and, if indicated, additional testing such as GeneXpert MTB/RIF assay (Cepheid, Sunnyvale, California, USA) if they could produce sputum. If indicated, additional testing also included ultrasound, computed tomography, and fine-needle aspiration/biopsy. Gastric aspirates and induced sputum procedures were not available. Children diagnosed with TB disease were started on treatment per the National TB Program guidelines of Pakistan [13].

### Data collection and measures

Demographic and clinical characteristics were collected and entered into a custom-built electronic data collection tool [11]. Demographic information included sex, age, weight, and the hospital where the child was enrolled for TB treatment. Weight-for-age percentile was recorded using WHO growth charts for 0–2 years old and the U.S. Centers for Disease Control and Prevention’s growth charts for children older than two years old [14]. Children were classified as under nourished if their weight was at or below the fifth percentile. Clinical characteristics included cough, duration of cough, fever, weight loss, presence of Bacille Calmette-Guérin (BCG) vaccination scars, exposure to TB within the past two years, and type of TB disease in the child (defined as any pulmonary involvement or only extrapulmonary) [15]. Facility-based characteristics were also included, such as whether the facility served a rural or urban population and whether it was a newly established TB treatment facility or not.

**Fig 2:**
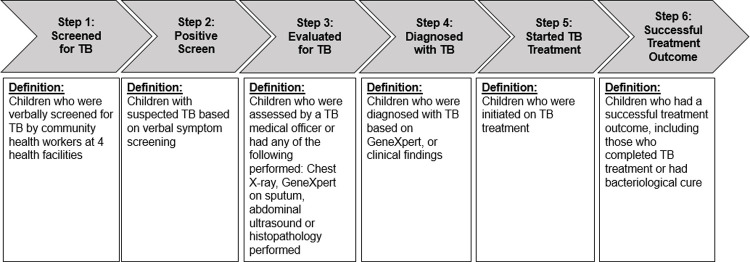
Steps in the childhood tuberculosis care cascade from screening to treatment outcome.

We combined process indicators from two cascade frameworks—proposed by the Zero TB Initiative [8]—to assess how the intensified patient-finding intervention performed, by age of the child screened. This modified cascade consists of six steps: (1) screened for TB, (2) positive screen, (3) evaluated for TB disease, (4) diagnosed with TB disease, (5) started treatment for TB disease, and (6) successful treatment outcome (Fig 2).

We used the following definitions to characterize how each child progressed through each step.

**Step 1. Screened for TB:** Children were classified as “screened for TB” if a health worker completed a verbal screening, which consisted of asking the child or caregiver (a) whether the child had contact with someone with TB in the last two years, or (b) whether the child presently had any of the following symptoms: glandular swelling, cough greater than two weeks, fever lasting two or more weeks, night sweats, and inappropriate weight loss (or failure to thrive).

**Step 2. Positive screen:** Children were classified as having a “positive screen” if they responded that they had contact with someone with TB in the last two years, had a cough greater than two weeks, had glandular swelling, and/or when two or more of the following symptoms were present: fever lasting two or more weeks, night sweats, and inappropriate weight loss (or failure to thrive).

**Step 3. Evaluated for TB:** Children were classified as “evaluated for TB” if they both (a) had a chest X-ray, Xpert MTB/RIF assay on sputum, abdominal ultrasound, or histopathology test performed, and (b) were assessed by a TB medical officer. Children who screen positive are referred to a clinician who conducts a clinical examination and takes a detailed medical history. A chest radiograph, complete blood count and erythrocyte sedimentation rate were done for all children and an Xpert MTB/RIF assay was done if the child could produce a sputum sample. Additional testing, included ultrasound, computed tomography, and fine-needle aspiration/biopsy, was done if extrapulmonary disease was suspected. Fig 1 includes the detailed flow of children through screening and diagnostic procedures.

**Step 4. Diagnosed with TB:** Children were classified as “diagnosed with TB” if a TB medical officer made a diagnosis of TB disease in the child upon review of the clinical, laboratory, radiological, and/or histopathological test results.

**Step 5. Started TB treatment:** Children were classified as “started TB treatment” if they initiated a regimen to treat TB disease.

**Step 6. Successful treatment outcome:** Children were classified as having a “successful treatment outcome” if they met the standard definitions for completing TB treatment or bacteriological cure [13, 16].

### Analysis

Children were included in this analysis if they were between 0–14 years of age. For each year of age, we calculated a percentage by dividing the number of children who completed each step of the cascade by those who were eligible for that step. We reported percentages at each age (by year of age) and—for each step in the cascade—we also calculated the average and standard deviation (SD) across the individual ages by year of age. We also calculated the cumulative percentage of children who completed all steps by multiplying the percentages who completed each step. We did this for each year of age from age 0 to 14 years, overall and by gender. Age-stratified data were not available for Step 1, so we calculated cumulative percentages among children who screened positive. We also calculated (among children who screened positive) the percentage of children with bacteriologic confirmation of TB disease.

We explored gaps by comparing the percentage of demographic and clinical characteristics of children who did or did not complete each step, using chi-squared tests. Analyses were complete in SAS version 9.4 (SAS Institute Inc., Cary, NC, USA).

### Ethics statement

The Institutional Review Board (IRB) of Interactive Research and Development, Karachi, Pakistan, reviewed and approved the original study protocol (approval number: IRD-IRB-2015-04-002). Verbal informed consent was obtained from all children’s guardians as well as from children over the age of seven. The subsequent analysis of de-identifiable data was determined to be non-human subjects research by the IRB of Harvard Medical School (approval number: HMS-IRB-20-0479).

## Results

During the study timeframe, 105,338 children 0–14 years old were verbally screened for TB disease. A total of 5,880 (5.6%) children had a positive TB screen. Of the 5,162 (87.8%) children who were subsequently evaluated for TB disease, 1,417 (27.5%) were diagnosed with TB disease, 1,404 (99.1%) initiated treatment for TB disease, and 1,311 (93.4%) experienced a successful treatment outcome. Of children diagnosed with TB disease, 714 (50.4%) were 0–5 years old, 799 (56.4%) were male, 248 (17.6%) had extrapulmonary TB, and 1,170 (84.2%) were undernourished.

**Fig 3:**
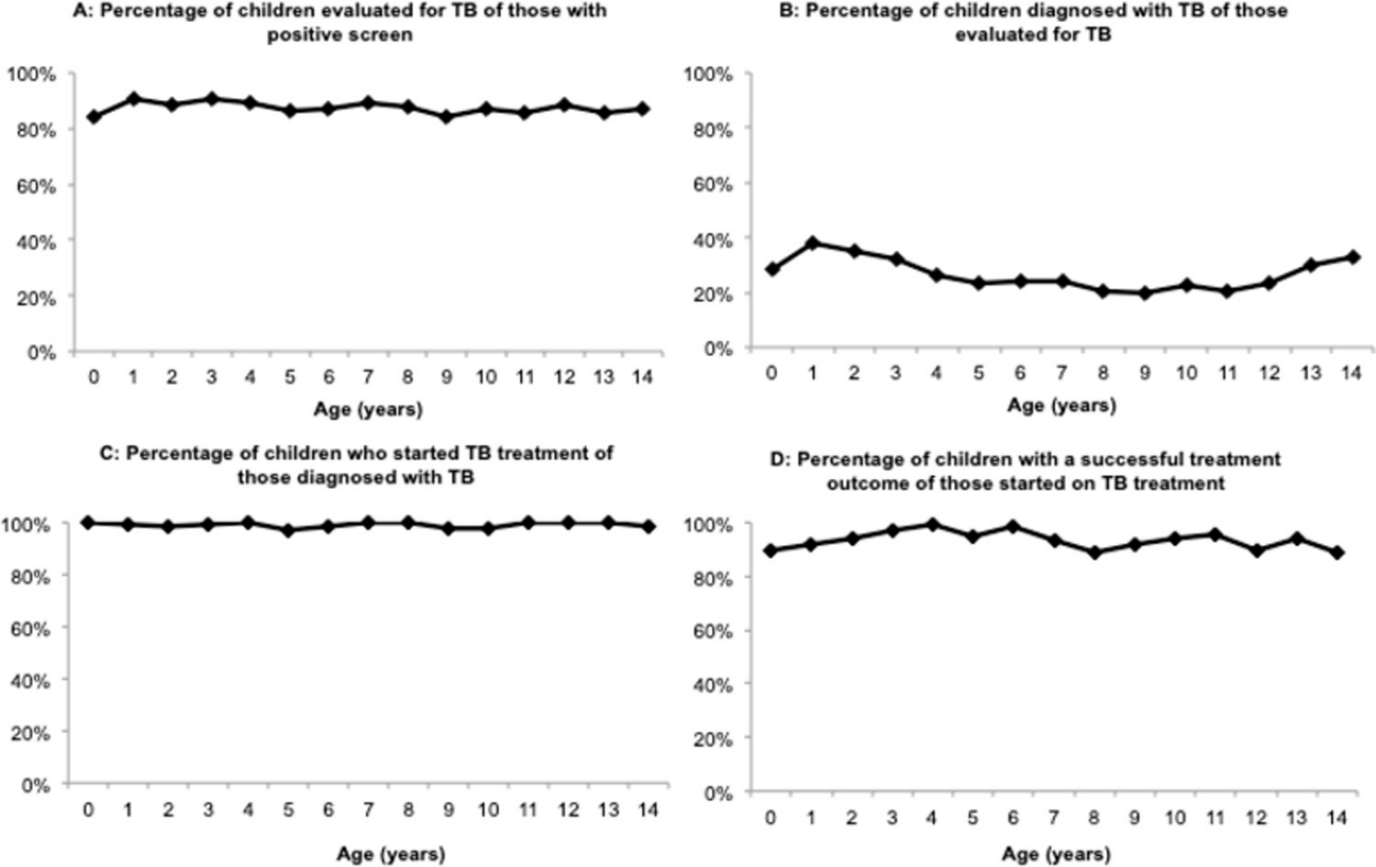
Percentage of children completing each step of the care cascade, by age.

We observed little variability by year of age in the percentage of children who had a complete evaluation, with an average across the individual age (by year) percentages of 87.5% (SD: 1.9%) (Fig 3, Panel A). The average percentage of children across each individual age who were diagnosed with TB disease was 26.8% (SD: 5.5%) (Fig 3, Panel B) and resulted in a non-linear trend: a higher percentage of children 0–4 (mean: 31.9%; SD: 4.8%), a decline in children 5–9 (mean: 22.4%; SD: 2.2%), and an increase in children 10–14 (mean: 26.0%; SD: 5.4%). The average of the individual age percentage of children who started treatment was relatively steady (mean: 99.1%; SD: 1.0%) (Fig 3, Panel C), while the average percentage for children who experienced a successful treatment outcome was more variable (mean: 93.3%; SD: 3.3%), as observed by an increase in early childhood and then a dip around age 8 and again in early adolescence (Fig 3, Panel D). Only 42 (3.0%) of children sick with TB had bacteriologic confirmation, with a sharp increase from ages 10 to 14.

**Fig 4:**
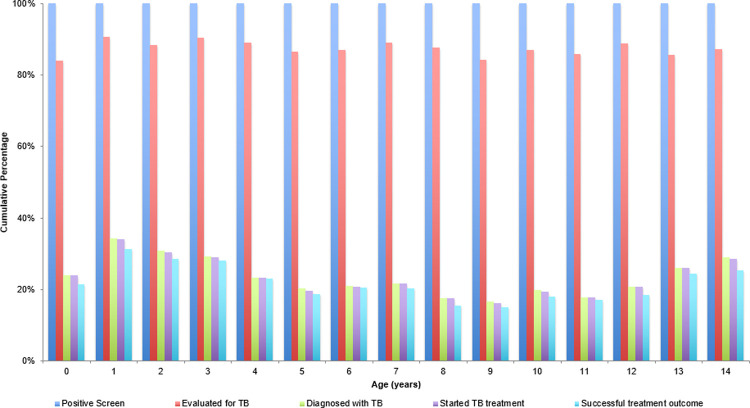
Cumulative percentages of all children with a positive screen completing each step of care cascade, by age.

The cumulative percentage and standard deviation—calculated among children who screened positive (Step 2)—was calculated for each year of age and then averaged across all ages. We observed that 87.5% (SD: 2.0%) of children were evaluated for TB disease after having a positive screen, 23.5% (SD: 5.3%) were diagnosed with TB disease after being evaluated, 23.3% (SD: 5.3%) were started on TB treatment after being diagnosed, and 21.7% (SD: 4.9%) experienced a successful treatment outcome (Fig 4).

Upon further investigation of the 12.5% of children who were not evaluated for TB despite a positive screen, we found they were less likely to have fever (p = 0.03), weight loss (p = 0.02), family history of TB (p<0.001), family members with TB who were sputum positive (p<0.001), and to be evaluated at a rural facility (p<0.001), compared to children who were evaluated for TB disease after a positive screen (Table 1).

**Table 1:** Characteristics of children who were evaluated for TB compared to children who were not evaluated for TB.

Variable		Children who completed an evaluation N = 5162		Children who did not complete an evaluation N = 718		*p*-value for chi-squared test
		n	%	n	%	
CHILD CHARACTERISTICS						
Female		2222	43.1	298	41.5	0.43
Age	0	397	7.7	75	10.5	0.13
	1	508	9.8	53	7.4	
	2	520	10.1	68	9.5	
	3	412	8.0	44	6.1	
	4	361	7.0	44	6.1	
	5	431	8.4	67	9.3	
	6	352	6.8	52	7.2	
	7	406	7.9	49	6.8	
	8	433	8.4	61	8.5	
	9	197	3.8	37	5.2	
	10	361	7.0	54	7.5	
	11	116	2.3	19	2.7	
	12	316	6.1	40	5.6	
	13	162	3.1	27	3.8	
	14	190	3.7	28	3.9	
Weight percentile ≤5		1589	97.3	27	100.0	0.38
Cough		1740	93.5	28	96.6	0.51
Cough Duration	No Cough	135	7.8	1	3.6	0.23
	<2 weeks	214	12.4	3	10.7	
	2–3 weeks	501	29.0	13	46.4	
	>3 weeks	881	50.9	11	39.3	
Fever		4064	78.9	540	75.2	0.03
Weight loss		3378	65.8	439	61.1	0.02
Presence of BCG scars		1037	59.1	11	39.3	0.04
Extra-pulmonary TB		248	17.6	0	0	N/A
FAMILY MEMBER CHARACTERISTICS						
Family history of TB		1432	27.7	4	0.6	<0.001
TB type in family is extra-pulmonary		15	1.1	0	0	1.00*
Family member with TB	Mother	1241	89.2	4	100.0	0.79
	Father	22	1.6	0	0	
	Other	128	9.2	0	0	
Family member was sputum positive		1247	90.1	4	44.4	<0.001
FACILITY CHARACTERISTICS						
Newly established TB treatment facility		459	8.9	61	8.5	0.73
Rural facility		754	14.6	67	9.3	<0.001

Abbreviations: BCG: Bacille Calmette-Guerin vaccine; TB: tuberculosis.

*Fisher’s exact test used; otherwise chi-squared test.

**Fig 5:**
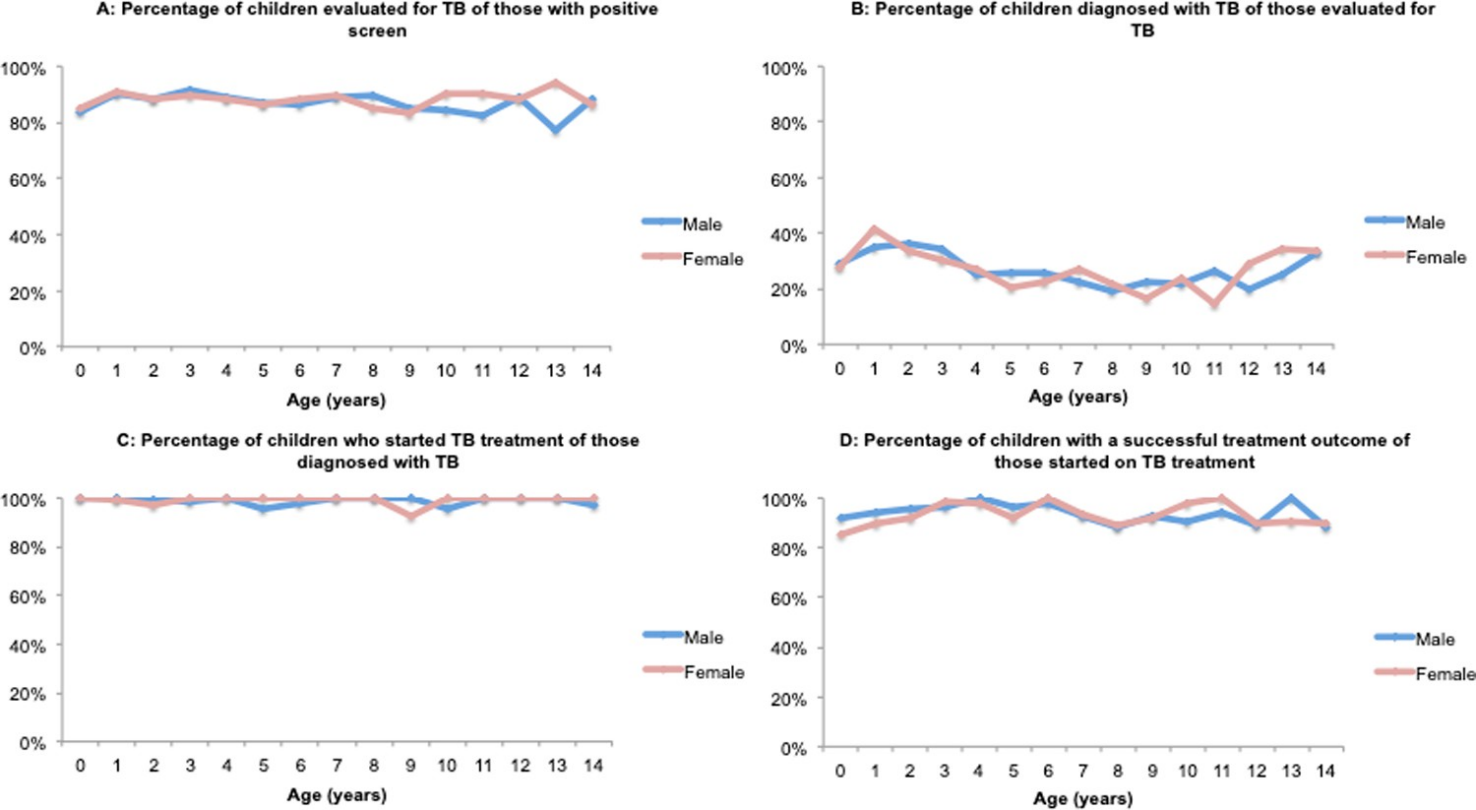
Percentage of children completing each step of the care cascade, by age and gender.

We observed little variability by gender across all ages (Fig 5). The percentage of males and females evaluated for TB was similar until age nine, at which point females trended only slightly higher (mean: 88.1%; SD: 2.7%) than males (mean: 86.7%; SD: 3.7%) (Fig 5, Panel A). The percentage of children diagnosed with TB showed similar variability for both genders, with an average of 26.7% (SD: 5.5%) for males and 26.8% (SD: 7.2%) for females (Fig 5, Panel B). The percentage of children who started treatment was steady across all ages, with an average of 98.9% (SD: 1.7%) among males and 99.2% (SD 2.0%) among females (Fig 5, Panel C). The percentage of children who had a successful treatment outcome was also similar for males and females with an average of 93.7% (SD: 4.0%) among males and 93.1% (SD: 4.5%) among females (Fig 5, Panel D).

## Discussion

We found that an intensified TB patient-finding intervention focused on children implemented in a high TB-burden setting performed with high effectiveness across all ages of the children screened. Delivery of care—including evaluating a child for TB, diagnosing TB, linkage to treatment, and treatment completion—were similar across ages. In particular, we found that a very high proportion of children sick with TB across all ages who were initiated on treatment subsequently completed treatment—93%—exceeding the World Health Organization’s End TB goal of 90% [17]. This is substantially higher than that identified by another care cascade evaluation conducted in children in Kenya and Uganda where only about 70% of children with TB completed treatment [18]. The effectiveness observed across all ages in this program sets a high standard for the quality of TB care that children can receive in high TB-burden areas.

The largest gap that we identified in this cascade—across children of all ages—was an average of 12.5% who screened positive but did not go on to receive a complete evaluation. Those not evaluated were significantly less likely to have a family history of TB, or be evaluated at a rural facility. Children presenting with fever or weight loss were significantly more likely to have further evaluation; fever indicated that there was some immediate underlying condition and provoked a prompt response from the evaluating medical officer and families [19]. The vast majority of children with a family history of TB received a complete evaluation, which is reflective of the concurrent implementation of a contact tracing intervention that aimed to capture contacts of TB patients diagnosed at the same facilities [20]. A previous study of this cohort of children with TB found that children diagnosed at rural facilities were more likely to experience successful treatment outcomes, pointing to potentially more established systems and trained staff in place that led to improved TB care for children [21].

Failure to diagnose TB disease is known to be a critical gap in the care of children with TB [17]. In this setting, we were unable to determine what proportion of all child cases was being missed by the diagnostic algorithm. With current tools, only a small proportion of children with TB will have bacteriologic confirmation; our observations are consistent with this limitation of existing tools, with a low yield of bacteriologic confirmation of disease and an increasing percentage with age. Notably, we were unable to determine whether the diagnostic percentage was above or below the true prevalence of disease in children in each age group. This program relied heavily on clinical diagnosis of TB disease, and sputum induction and gastric aspirate samples were not available. This experience highlights the importance of TB history, physical exam, contact and exposure history, and evidence from a chest radiograph when diagnosing children with TB [22]. It also points to the need for more accurate clinical prediction tools to capture children for whom current TB tests will consistently fail to obtain bacteriologic confirmation but who require prompt initiation of life-saving treatment [23, 24].

We observed that 56% of children diagnosed with TB were male; this contrasts with two studies from Pakistan where more females had TB disease and large gender gaps were observed [25, 26]. Unfortunately, gender data were not collected at our initial screening step, so we are unable to determine if more male children were being brought to the health facilities and subsequently screened for disease. No difference was observed between males and females with regard to treatment outcomes. An analysis of a similar intensified patient-finding program in Pakistan found that males were at higher risk for unsuccessful treatment outcomes than females [25]. In contrast, this patient-finding intervention demonstrated great gender parity in effectiveness at each step of the cascade.

There was a high TB yield in younger children, with over half of those cases diagnosed being <5 years old. This intervention conducted equitable screening across all ages of children and was rolled out in public hospitals that also provided post-natal child care and early immunization. Thus, in this program, the fact that the youngest children—who are typically disproportionately missed—contributed most of the TB cases detected shows that this type of strategy is effective in closing detection gaps. With two-thirds of pediatric cases unreported or undiagnosed globally [3–5], it is essential to identify effective strategies to close this broader case detection gap [27]. While improving contact management programs will be important, the majority of children who fall sick with TB will not be identified as household contacts [28, 29]. Programs will need to include other child-centered strategies, such as intensified patient-finding interventions in health facilities that employ child-specific diagnostic algorithms. The cascade framework we used is a tool for self-evaluation of gaps in care and can serve as a common language for mutual exchange and shared learning, with the identification of age- or gender-specific gaps that can inform further tailored strategies.

A major strength of our study is the large cohort size which allowed for assessment by individual age- and gender-strata. Other reports of pediatric patient-finding interventions—where the steps of the care cascade were evaluated—examined children as a single group (0–14 years), not disaggregated by age [18]. Further, another strength of our study is the combination of the “search” and “treat” cascades.

Our study has several limitations. First, age-stratified data were not collected at the moment the children were screened, precluding an age-stratified analysis of that first cascade step. This would have provided important information regarding who reached the facilities and then screened positive. Second, the use of intensified patient-finding interventions may lead to concerns about over-diagnosis. Notably, this program included a training component for local staff on childhood TB diagnosis, outcomes, and recording, and a robust monitoring and evaluation system built into the intervention with support from the TB Program of Sindh Province; the goal was to ensure that the diagnostic algorithm was followed, over-diagnosis was minimized, and linkage to treatment was ensured.

It is also worth highlighting the risk of misinterpretation of the cumulative percentage that we report for completion of the full cascade. Because age was not recorded at the moment that the children were screened, these cumulative percentages were only calculated starting at Step 2, namely, among children who screened positive. This explains why >20% of that cohort was diagnosed with TB disease and completed treatment. Had we started from the >105,000 children screened, the cumulative percentage completing treatment would be around 1.2%. This order of magnitude difference reflects which step—either those screened at all, or those who screened positive—has data available for analysis. We were able to use age-stratified data to observe the cumulative percentage of children who completed treatment completion among those who screened positive, not among all those who were screened. Both are useful benchmarks for program teams as they estimate the numbers of children who will require TB treatment among those who are targeted for screening.

A care cascade framework can be used to evaluate intensified TB patient-finding programs to understand how they perform for specific groups. In Pakistan, we found that an intensified case detection program was effective for children across ages 0–14 years. The care cascade indicators can identify gaps in TB detection, treatment initiation, and treatment outcomes, and these can be compared by age. Variability in effectiveness by age could inform tailored interventions and programs, including maternal and child health programs or treatment support programs, to identify strategies to close gaps in pediatric TB care and management.
